# Demonstrative Midwifery

**Published:** 1850-05

**Authors:** 


### ARTICLE VI.

### DEMONSTRATIVE MIDWIFERY.

We observe by our contemporary, the Buffalo Med. & Surg. Journal, that a Demonstration of Natural Labour, was made by Prof. White before the medical class, in the University of Buffalo last winter; and that the affair went off very satisfactorily to all parties concerned. Although in the present state of society in America it is not probable that such a means of instruction will become general, or often be repeated. Still, we unhesitatingly say, that when considered properly, it is a false modesty that condemns it.

